# Supplementary material for: Humanoid robots to mechanically stress human cells grown in soft bioreactors
Source: Commun Eng. 2022 May 26;1:2. doi: 10.1038/s44172-022-00004-9 (PMC10938861; doi:10.1038/s44172-022-00004-9)
Supplement: Supplementary file 1 — Supplementary information [file 44172_2022_4_MOESM1_ESM.pdf]

Supplementary information

Supplementary Table 1: List of materials used in the soft chamber fabrication.

Chamber component	Material	Source
Scaffold	Medical grade PCL (IV: 1.4-2.0 dl/g)	Resin: Ashland Specialities Ireland, Tallaght, Ireland
Membrane	Polyurethane (TFL-2EA, 50µm)	Permali Gloucester Limited, Goucester, UK
3DP inserts	Polyamide 12 (PA2200, White)	EOS GmbH - Electro Optical Systems, Krailling, Germany
O-rings	EPDM rubber (70 ShA FDA Peroxide Cured)	Polymax, Bordon, UK
Tubing	PTFE (1/16 OD, ID 0.8mm)	Sigma Aldrich, Dorset, UK
Resin	Room temperature curing epoxy (Epotek 301)	Epoxy Technology Inc., Billerica, USA
Screws	Stainless steel (M2 x 10 Slot cheese machine screw DIN 84 A2)	Precision Technology Supplies Ltd, East Grinstead, UK
Cord loop	10lb-300lb PE braided cord	Hercules, China

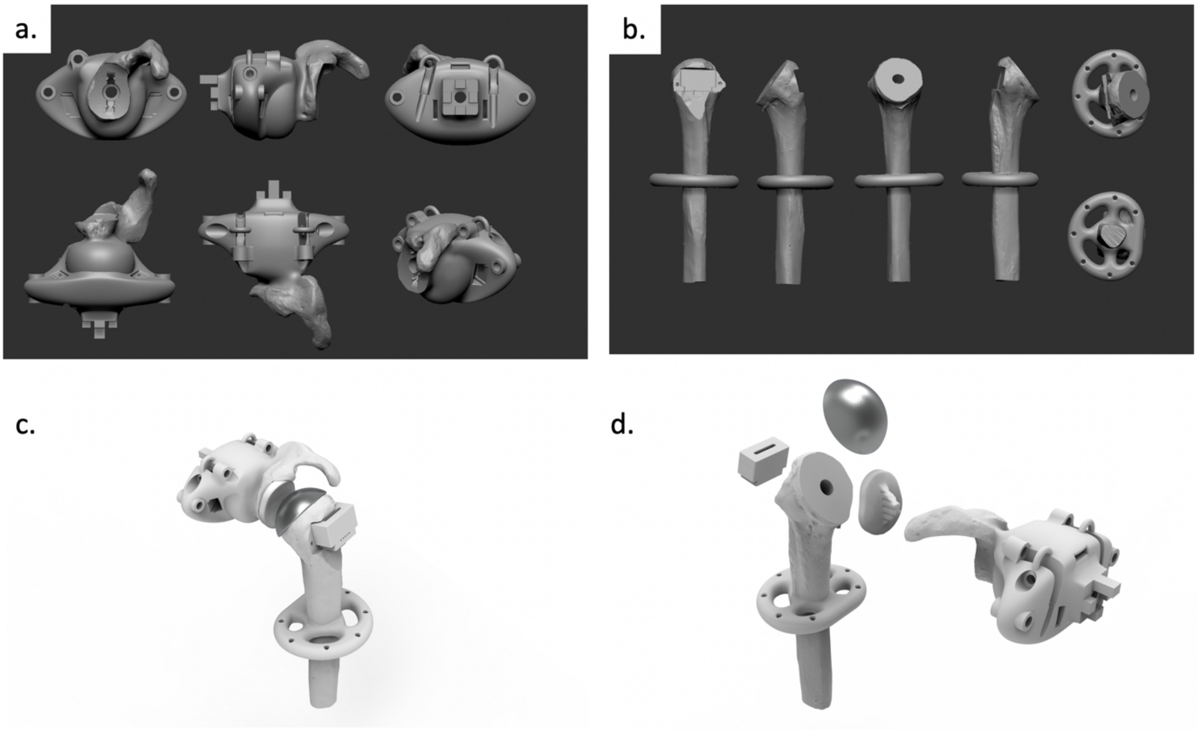

Supplementary Figure 1. Details of the new shoulder structure. a. Humanoid shoulder base modified to include the glenoid and coracoid process. b. Proximal part of the humerus modified with a ring for the attachment of the muscle string and with the insert imprint for the chamber attachment. c. View of the assembly with a shoulder implant and the lower insert of the bioreactor chamber. d. Exploded view showing the details of the assembly from the front view.

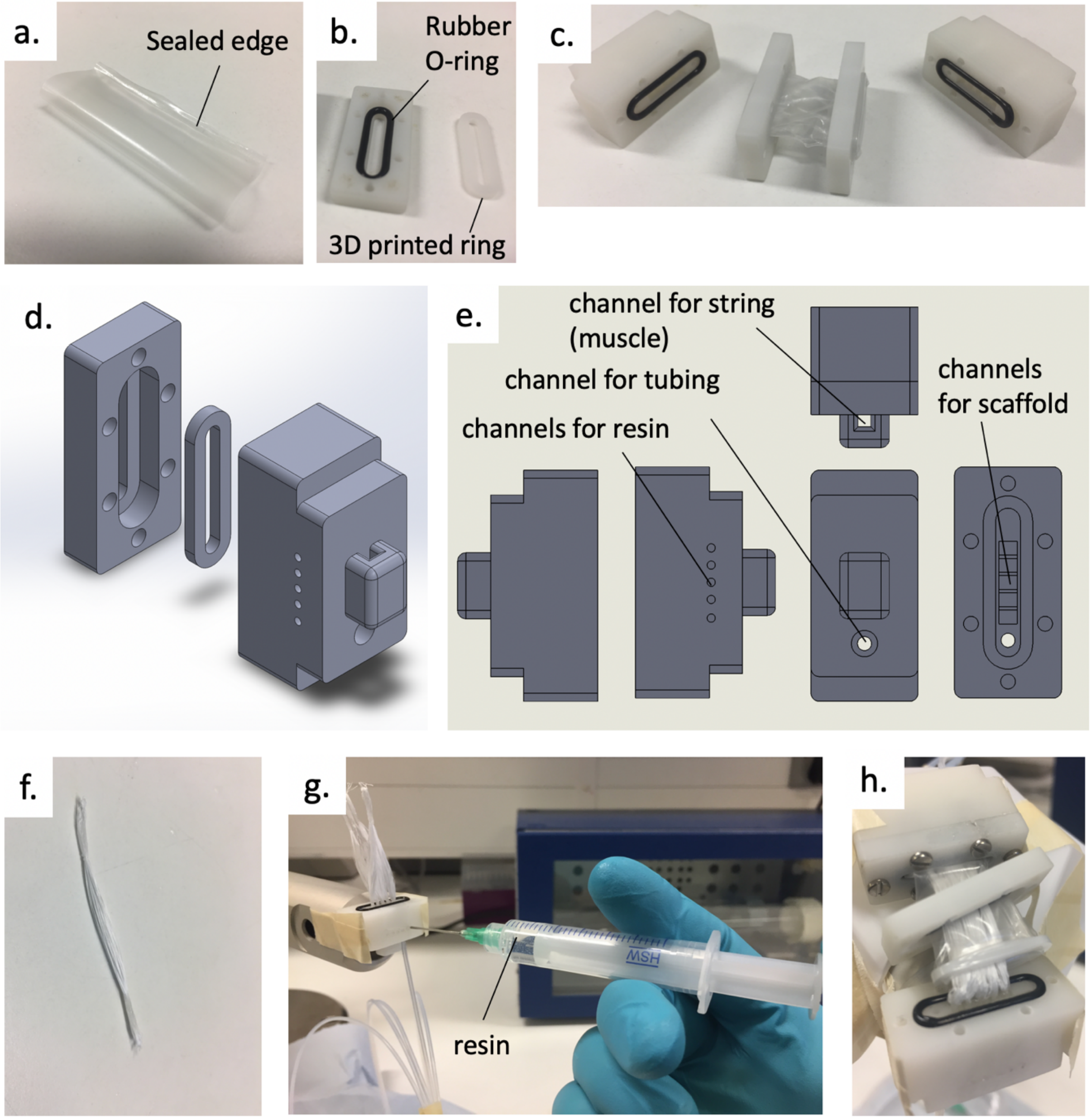

Supplementary Figure 2. Chamber components and assembly. a. The membrane was made of a thin sheet of transparent polyurethane rolled into a tubular shape and sealed along the long edge. b. 3D printed end plate and ring used to secure the membrane. c. The membrane is passed in and then out of the 3D printed ring, itself fitted in the end plate. d. overview showing how the 3D printed component fit together. e. Detail of the main insert body showing the different channels created for the resin, tubing, muscle string and scaffold. f. Bundle made of 40 electrospun filaments to be inserted in a scaffold channel. g. Injection of the resin to secure the bundles in the main insert. h. View of the chamber before its closure with screws.

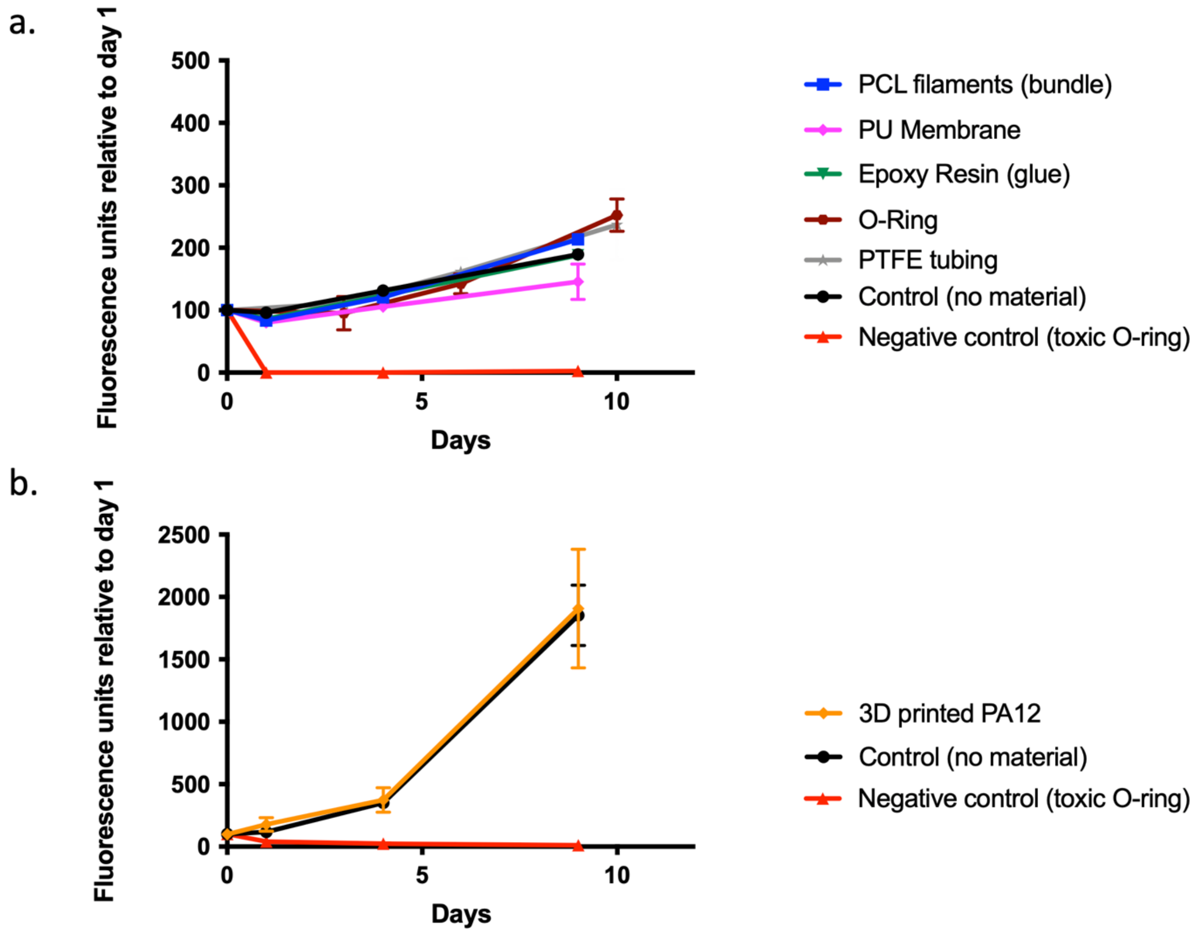

Supplementary Figure 3. Biocompatibility of the different materials in the chamber assembly that are exposed to the culture medium during use. Cells were seeded at a density of 5000 cells per 24-well and material fragments were directly added to the medium following the first Presto assay measurements at day 0. Cells were then cultured in presence of the different components for a period of 9 days: a. all materials except PA12 inserts, b. 3D printed PA12 inserts. Controls included a ‘no material’ control (medium only) and a cytotoxic control (nitrile rubber O-ring). Error bars represent standard deviation (n=3 biologically independent samples, with samples in triplicates). The data indicates that none of the chamber materials showed signs of toxicity to the cells.

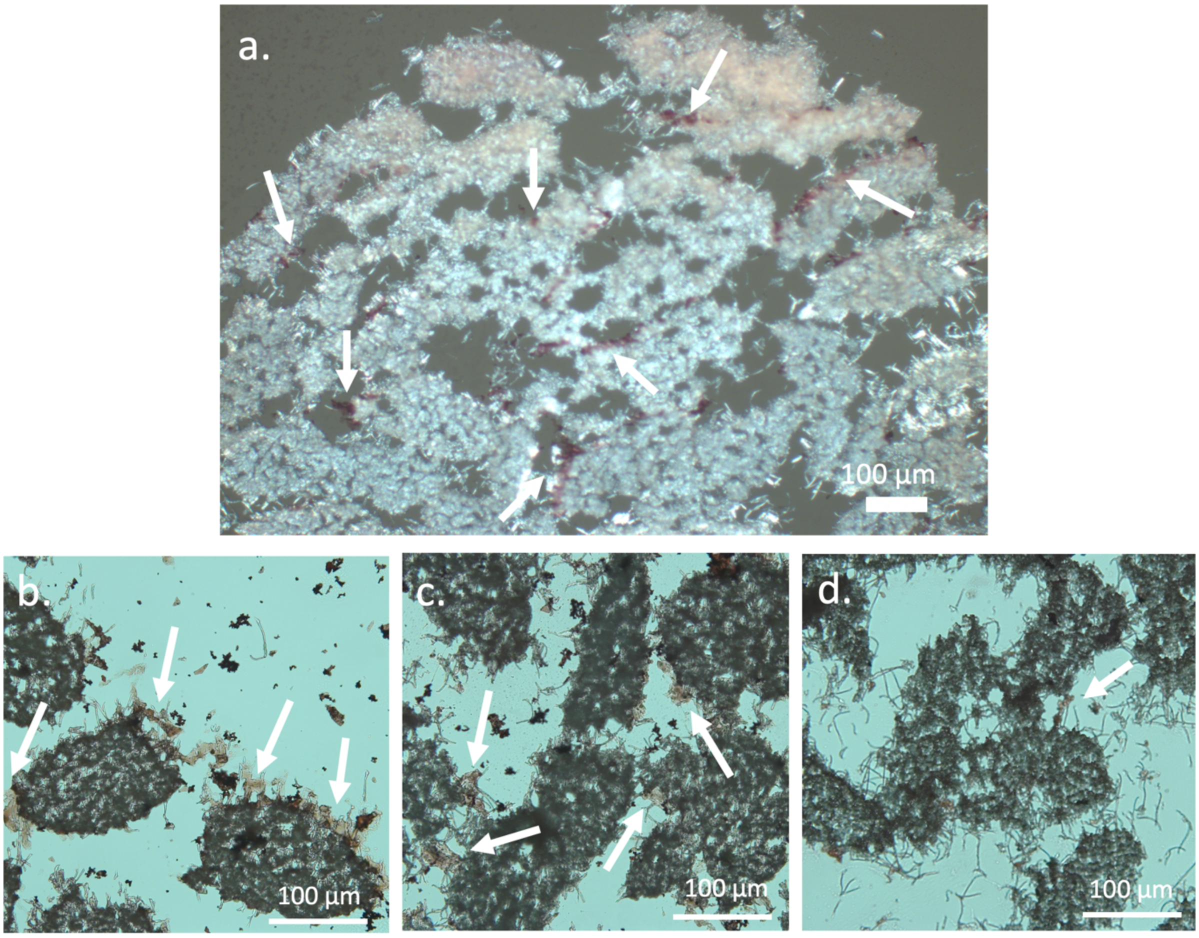

Supplementary Figure 4. Stained cryosections of the cell-material constructs: a. Sample at day 1 showing that cells (dark red areas highlighted with white arrows) are seeded within the scaffold material, not just at its surface (top arc), b-d: Samples collected after 14 days of culture under different loading conditions showing biological/gel material (white arrows) at the surface of the electrospun filaments: static (b), LFR (c) and HFR (d). The amount of stained material observed in these sections was consistent with the fluorescence microscopy data.

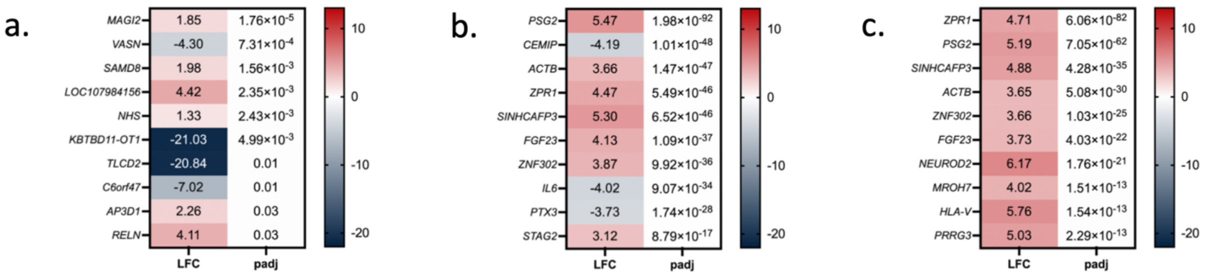

Supplementary Figure 5. Heatmaps showing the top 10 differentially expressed genes of human fibroblasts cultured under different loading regimes for 14 days, including (a) Static vs LFR, (b) Static vs HFR, and (c) LFR vs HFR. LFC and adjusted p-value (padj) with upregulated genes in red and downregulated genes in blue. For instance, *ACTB*, a gene that encodes for the cytoskeletal protein beta-actin, was significantly upregulated in HFR compared to static and LFR samples, suggesting changes to the cell cytoskeleton under HFR conditions (static: n=3; LFR, HFR: n=2).

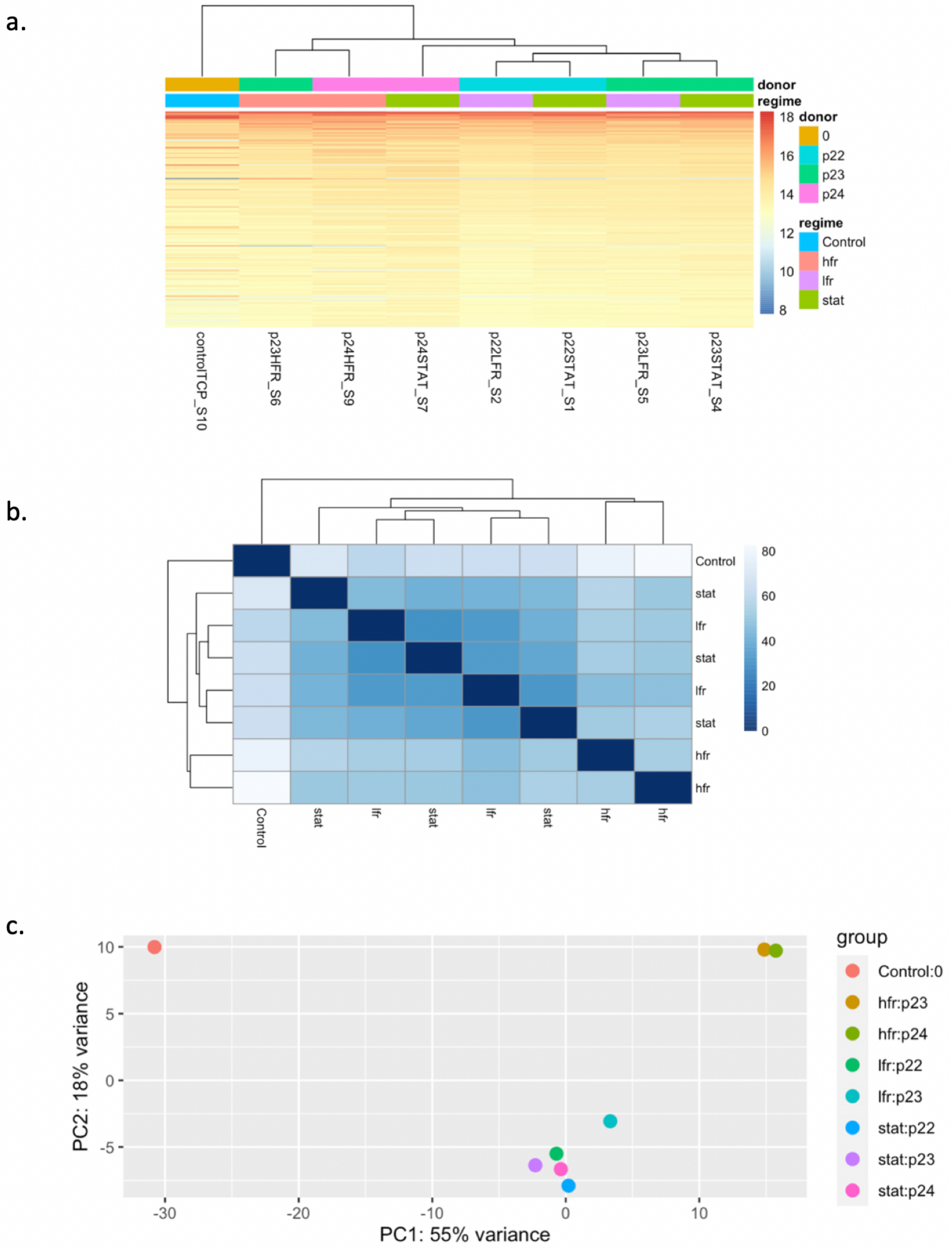

Supplementary Figure 6. Overview of different plots showing clustering of the fibroblasts samples in the bulk RNA-Seq analysis: (a) Heatmap of the counts matrix, blue shows low counts while red shows high counts. (b) Heatmap of the sample-to-sample distances, dark blue represents no to little difference while white represents large differences between samples. (c) Principle component (PCA) plot for chondrocytes samples. Different sample types are represented with different colours.
