# Supplementary material for: Humanoid robots to mechanically stress human cells grown in soft bioreactors
Source: Commun Eng. 2022 May 26;1:2. doi: 10.1038/s44172-022-00004-9 (PMC10938861; doi:10.1038/s44172-022-00004-9)
Supplement: Supplementary file 7 — Description of Additional Supplementary Files [file 44172_2022_4_MOESM7_ESM.pdf]

Description of Additional Supplementary Files

File Name: Supplementary Movie 1

Description: Range of motion of the original shoulder developed by Devanthro

File Name: Supplementary Movie 2

Description: Tensile test to failure of the soft bioreactor chamber (video accelerated 10x) the column to the right.

File Name: Supplementary Movie 3

Description: High force regime (HFR) applied during culture with the humanoid bioreactor system

File Name: Supplementary Movie 4

Description: Low force regime (LFR) applied during culture with the humanoid bioreactor system
