# Supplementary material for: Humanoid robots to mechanically stress human cells grown in soft bioreactors
Source: Commun Eng. 2022 May 26;1:2. doi: 10.1038/s44172-022-00004-9 (PMC10938861; doi:10.1038/s44172-022-00004-9)
Supplement: Supplementary file 8 — Reporting Summary [file 44172_2022_4_MOESM8_ESM.pdf]

nature portfolio

Corresponding author(s): Pierre-Alexis Mouthuy

Last updated by author(s): 15/03/2022

Reporting Summary

Nature Portfolio wishes to improve the reproducibility of the work that we publish. This form provides structure for consistency and transparency in reporting. For further information on Nature Portfolio policies, see our Editorial Policies and the Editorial Policy Checklist.

Statistics

For all statistical analyses, confirm that the following items are present in the figure legend, table legend, main text, or Methods section.

n/a	Confirmed	
☐	☒	The exact sample size (n) for each experimental group/condition, given as a discrete number and unit of measurement
☐	☒	A statement on whether measurements were taken from distinct samples or whether the same sample was measured repeatedly
☐	☒	The statistical test(s) used AND whether they are one- or two-sided<br>*Only common tests should be described solely by name; describe more complex techniques in the Methods section.*
☐	☒	A description of all covariates tested
☐	☒	A description of any assumptions or corrections, such as tests of normality and adjustment for multiple comparisons
☐	☒	A full description of the statistical parameters including central tendency (e.g. means) or other basic estimates (e.g. regression coefficient) AND variation (e.g. standard deviation) or associated estimates of uncertainty (e.g. confidence intervals)
☒	☐	For null hypothesis testing, the test statistic (e.g. F, t, r) with confidence intervals, effect sizes, degrees of freedom and P value noted<br>*Give P values as exact values whenever suitable.*
☒	☐	For Bayesian analysis, information on the choice of priors and Markov chain Monte Carlo settings
☒	☐	For hierarchical and complex designs, identification of the appropriate level for tests and full reporting of outcomes
☒	☐	Estimates of effect sizes (e.g. Cohen's d, Pearson's r), indicating how they were calculated

Our web collection on statistics for biologists contains articles on many of the points above.

Software and code

Policy information about availability of computer code

Data collection	Only commercial software were involved in data collection:<br>- CAD softwares: Autodesk Fusion (San Francisco, United States) and Solidworks (edition 2016, Dassault Systèmes SolidWorks Corporation, Waltham, USA)<br>- TestXpert II for test to failure (Zwick Roell Group, Ulm, Germany)<br>- High-speed data logger software for force sensor (model 9330-MSDI-IP43, Interface Force Measurements Ltd)<br>- Fluorostar Omega microplate reader software (BMG Labtech, Ortenberg, Germany)<br>- Illumina software CASAVA v1.8 to generate Raw FASTQ files<br>- Graphpad PRISM version 7 software (GraphPad Software Inc., La Jolla, CA, USA) adn GraphPad Prism 9.2.0 (GraphPad Software, La Jolla, CA, USA).
Data analysis	Raw FASTQ files were processed using workflows developed by the Cribbs's lab (https://github.com/cribbslab) as specified in the manuscript.

For manuscripts utilizing custom algorithms or software that are central to the research but not yet described in published literature, software must be made available to editors and reviewers. We strongly encourage code deposition in a community repository (e.g. GitHub). See the Nature Portfolio guidelines for submitting code & software for further information.

Data

Policy information about availability of data

All manuscripts must include a data availability statement. This statement should provide the following information, where applicable:

- Accession codes, unique identifiers, or web links for publicly available datasets
- A description of any restrictions on data availability
- For clinical datasets or third party data, please ensure that the statement adheres to our policy

Datasets and CAD files are available from the authors and are deposited on the University of Oxford's institutional repository, ORA-Data (DOI: 10.5287/bodleian:9eX14oddB). The open-source designs of Devanthro's Roboy Project can be found at https://devanthro.com/technology/.

Field-specific reporting

Please select the one below that is the best fit for your research. If you are not sure, read the appropriate sections before making your selection.

☒ Life sciences ☐ Behavioural & social sciences ☐ Ecological, evolutionary & environmental sciences

For a reference copy of the document with all sections, see nature.com/documents/nr-reporting-summary-flat.pdf

Life sciences study design

All studies must disclose on these points even when the disclosure is negative.

Sample size	Given the novelty of the work, both the cost of experiments and complexity of the setup were limiting factors with regard to sample size. Each chamber required its own perfusion system (tubing, gas exchanger, reservoir, etc.), while one robotic arm was available to carry out the work, meaning that it was not possible to handle a high number of repeats at the same time. As a result, only 1 biological sample for each condition (static, HFR and LFR), i.e. 3 chambers, could be included in each experimental repeat.
Data exclusions	No data was excluded from the analyses, excepted for the bulk RNA work where, as disclosed in the manuscript, two samples had to be excluded due to high percentage of over-represented sequences and low alignment scores.
Replication	The demonstration of the humanoid bioreactor with human cells involved 4 experimental repeats.
Randomization	No randomization was necessary at this stage
Blinding	No blinding was necessary at this stage

Reporting for specific materials, systems and methods

We require information from authors about some types of materials, experimental systems and methods used in many studies. Here, indicate whether each material, system or method listed is relevant to your study. If you are not sure if a list item applies to your research, read the appropriate section before selecting a response.

Materials & experimental systems

n/a	Involved in the study	
☒	☐	Antibodies
☐	☒	Eukaryotic cell lines
☒	☐	Palaeontology and archaeology
☒	☐	Animals and other organisms
☒	☐	Human research participants
☒	☐	Clinical data
☒	☐	Dual use research of concern

Methods

n/a	Involved in the study	
☒	☐	ChIP-seq
☒	☐	Flow cytometry
☒	☐	MRI-based neuroimaging

Eukaryotic cell lines

Policy information about cell lines

Cell line source(s)	Human dermal fibroblast cell line (HFF-1, ATTC, Manassas, Virginia)
Authentication	The cell line was not tested for authentification
Mycoplasma contamination	Cells were regularly checked for Mycoplasma infection using a MycoAlert testing kit (Lonza, UK), according to the

Mycoplasma contamination	manufacturer's instructions. None of the samples used in the study showed signs of contamination.
Commonly misidentified lines<br>(See ICLAC register)	NA
